# Exploring the Relationship between Epicardial Fat Thickness and Coronary Revascularization: Implications for Cardiovascular Health

**DOI:** 10.3390/jcm13010247

**Published:** 2023-12-31

**Authors:** Antonino Davide Romano, Antonella La Marca, Rosanna Villani, Moris Sangineto, Vincenzo Manuppelli, Natale Daniele Brunetti, Gianluigi Vendemiale, Gaetano Serviddio

**Affiliations:** 1 Internal Medicine and Liver Unit, Department of Medical and Surgical Sciences, University of Foggia, Policlinico Riuniti 71122 Foggia, Italy; antonella89la.marca@gmail.com (A.L.M.); rosanna.villani@unifg.it (R.V.); moris.sangineto@unifg.it (M.S.); gaetano.serviddio@unifg.it (G.S.); 2 Cardiology Unit, Department of Medical and Surgical Sciences, University of Foggia, Policlinico Riuniti, 71122 Foggia, Italy

**Keywords:** epicardial fat, EAT, echocardiography, Coronary Artery Disease, PTCA

## Abstract

Background: this study aimed to assess the complex relationship between EAT thickness, as measured with echocardiography, and the severity of coronary artery disease (CAD). We investigated whether individuals with higher EAT thickness underwent coronary revascularization. Subsequently, we conducted a three-year follow-up to explore any potential modifications in EAT depots post-angioplasty. Methods: we conducted a prospective and retrospective cross-sectional observational study involving 150 patients consecutively referred for acute coronary syndrome, including ST-elevation myocardial infarction (STEMI), non-ST elevation myocardial infarction (NSTEMI), and unstable angina. Upon admission (T0), all patients underwent coronary angiography to assess the number of pathologic coronary vessels. Percutaneous transluminal coronary angioplasty (PTCA) was performed based on angiogram results if indicated. The sample was categorized into two groups: non-revascularized (no-PTCA) and revascularized (PTCA). Transthoracic echocardiograms to measure epicardial fat thickness were conducted at admission (T0) and after a 3-year follow-up (T1). Results and conclusions: findings revealed a positive correlation between EAT thickness and the severity of coronary artery disease (CAD), with patients undergoing PTCA showing decreased EAT thickness after three years. Echocardiography demonstrated reliability in assessing EAT, offering potential for risk stratification. The study introduces a cut-off value of 0.65 cm as a diagnostic tool for cardiovascular risk. Incorporating EAT measurements into clinical practice may lead to more precise risk stratification and tailored treatment strategies, ultimately reducing the burden of cardiovascular disease.

## 1. Introduction

The adipose tissue is a highly different and dynamic organ characterized not only by different types of tissue (white, brown and brite) and distribution across multi-depot but also by its plasticity, allowing it to alter its structural, cellular and molecular characteristics in response to various conditioning and regulatory factors in both physiological and pathological microenvironments [1]. White adipose tissue (WAT) can be broadly classified based on its location, primarily as subcutaneous adipose tissue (SAT), which is situated beneath the skin, and visceral adipose tissue (VAT), which surrounds the inner organs in the abdominal cavity and mediastinum. However, when the storage capacity of SAT is exceeded or its ability to generate new adipocytes is impaired, fat begins to accumulate in areas outside the subcutaneous tissue, particularly in VAT [2]. The role of VAT in increasing cardiovascular risk compared to peripheral (subcutaneous) adipose tissue has been well established [3], renewing scientific interest in the contribution of organ-specific adiposity to the pathophysiology of cardiometabolic diseases.

We focused our attention on epicardial adipose tissue (EAT), the specific VAT depot located around the heart. Despite being considered a useless and irrelevant innocent bystander for many years, the active role of epicardial fat in both physiological and pathological cardioprotective mechanisms is not well established [4]. Pericardial fat is situated along the outer layer of the fibrous pericardium, while EAT is located between the myocardium and the visceral pericardium. Importantly, there are several distinctions between epicardial and pericardial fat. Firstly, they originate from different embryological sources, with epicardial fat derived from the splanchnopleuric mesoderm and pericardial fat from the thoracic mesenchyme. Secondly, epicardial fat primarily consists of small adipocytes and exhibits mixed cellularity with stromal preadipocytes, fibroblasts, macrophages, mast cells and lymphocytes. In contrast, pericardial fat is composed of larger, mature adipocytes and pro-inflammatory cells [5,6]. The first hypothesis proposing a causal role of EAT in the development of coronary atherosclerosis was formulated by Burnsides et al. in 1950, who observed that atherosclerotic lesions were preferentially localized in coronary arteries surrounded by adipose tissue [7]. Such observation was subsequently demonstrated by several authors [8]. Coronary artery disease (CAD) is a dynamic process involving the accumulation of atherosclerotic plaques and functional alterations in coronary circulation. Adverse cardiometabolic conditions, such as chronic and long-term ischemic circumstances typically seen in CAD, can lead to a shift in EAT phenotype and biology, resulting in a pro-inflammatory profile [9].

While MRI is currently regarded as the gold standard for measuring both the thickness and volume of epicardial fat, its high costs and lengthy examination times limit its clinical utility. Cardiac CT, with its superior spatial resolution compared to both MRI and ultrasonography, offers improved sensitivity and accuracy in assessing epicardial fat. However, its high costs and, more importantly, exposure to ionizing radiation, currently restrict its usage [10]. Transthoracic ultrasound is the most used imaging modality for assessing epicardial fat thickness, primarily due to its cost-effectiveness, safety, ease of execution and reproducibility. It can be conveniently utilized in outpatient settings. However, the initial studies on this subject lack comparability due to the absence of a standardized method for echocardiographic measurement, particularly regarding location and phase of the cardiac cycle. The technique for measuring epicardial fat thickness with transthoracic ultrasound was developed and validated by the Iacobellis et al. [11]. Although there is currently no consensus on the cutoff value distinguishing physiological from pathological epicardial fat thickness, recent evidence suggests that a thickness greater than 5 mm (or a volume exceeding 125 mL or 68 mL/m^2^) can be considered abnormal [12]. The volume of epicardial fat, measured using CT, correlates with other biomarkers such as the coronary artery calcium score (CAC), which in turn is a predictor of CAD. This association seems to be independent of other cardiovascular risk factors [13]. Although the correlation between epicardial fat thickness and severity of coronary artery disease (CAD) has been demonstrated, systematic utilization of this data is still not feasible due to the costs and time requirements associated with diagnostic techniques such as MRI and CT.

In our investigation, we aimed to elucidate the intricate relationship between epicardial adipose tissue (EAT) and coronary atherosclerosis, with a specific focus on patients presenting with acute coronary syndrome. The primary objective was to examine whether individuals with greater EAT thickness underwent coronary revascularization and to explore the potential modifications in EAT depots three years post-angioplasty. The rationale for our study was rooted in the hypothesis that the extent of epicardial fat accumulation might correlate with the severity of coronary artery disease (CAD). To address this, we conducted a prospective cross-sectional observational study, employing echocardiographic measurements of epicardial fat thickness to predict CAD severity. Importantly, we sought to leverage the dynamic interplay between EAT and coronary tissue to assess the efficacy of angioplasty as a therapeutic intervention. Over a three-year period, we tracked the enrolled patients to measure the quantity of epicardial fat, aiming to discern whether angioplasty could indeed modify EAT depots and, if so, to understand the underlying mechanisms driving these modifications. By focusing on patients with acute coronary chest pain and investigating the impact of coronary revascularization on EAT thickness, our study provides valuable insights into the potential interventional effects on epicardial fat and its implications for cardiovascular health.

## 2. Materials and Methods

### 2.1. Study Setting

From 2018 to 2020, we conducted a prospective and retrospective cross-sectional observational study on individuals referred for acute coronary syndrome, that is, ST-elevation myocardial infarction (STEMI), non-ST elevation myocardial infarction (NSTEMI), and unstable angina (NSTEMI). The study involved a cohort of 150 patients consecutively admitted to the Cardiovascular Intensive Care Unit of Policlinico Riuniti (Foggia). All patients required coronary angiography, and if deemed necessary, underwent percutaneous transluminal coronary angioplasty (PTCA) during the procedure.

The inclusion criteria for the study encompassing subjects who were suspected cases of CAD, including symptomatic patients with an intermediate pretest probability of CAD or those with uninterpretable electrocardiograms or unable to exercise during stress tests. Patients presenting with any constellation of clinical findings consistent with CAD, including symptoms such as atypical chest pain, chest tightness, epigastric pain/burning, jaw/shoulder pain, dyspnea, worsening/reduced effort tolerance, new ECG abnormalities, and unequivocal stress tests. Prior to participation, all individuals provided written informed consent to be part of the study. Exclusion criteria: patients with a previous history of coronary artery bypass grafting surgery, history of percutaneous coronary intervention, known pericardial disease, current or recent history of pericardial effusion, were excluded.

These inclusion and exclusion criteria were established to ensure the enrollment of a specific population of suspected CAD cases and to maintain homogeneity within the study cohort.

Upon admission (T0) and after a follow-up period of three years (T1), each participant underwent transthoracic two-dimensional guided M-mode echocardiography using a Philips Affiniti 70 system, following standard techniques, with subjects positioned in the left lateral decubitus posture. The echocardiograms were recorded on videotape for later analysis. A single echocardiographer, who was blinded to the patients’ data, reviewed and reported the echocardiographic findings [14].

To conduct the echocardiographic study, we meticulously recorded 10 cardiac cycles of two-dimensional parasternal long- and short-axis views, as well as 10 cycles of M-mode imaging, ensuring the optimal orientation of the cursor beam in each view [15]. Specifically, we focused on measuring the epicardial fat thickness, defined as the echo-free space between the myocardium and visceral pericardium, on the free wall of the right ventricle, employing both the parasternal long- and short-axis views at end-diastole [16]. We deliberately selected the right ventricle for epicardial fat measurement, as this location has been demonstrated to exhibit the highest absolute epicardial fat layer thickness [17].

The severity of coronary atherosclerosis were derived from the SYNTAX score [18], which is commonly employed to assist the clinician in selecting the optimal revascularization strategy, aiming for the best possible outcome for the patient. The SYNTAX score is calculated during the coronary angiographic procedure. Since it is primarily based on two criteria, namely the number of involved coronary arteries and the degree of stenosis, for our objective, we have arbitrarily chosen to utilize the number of involved coronary arteries to stratify the severity of coronary artery disease [18,19]. The number of stenotic major coronary arteries (left anterior descending artery, circumflex artery, and right coronary artery) determined the classification as 1-, 2-, or 3-vessel disease.

### 2.2. Statistical Analysis

Continuous variables were expressed as mean ± standard deviation, while categorical variables were presented as frequencies and percentages. Categorical variables and continuous variables were compared with Pearson’s χ2 test or Fisher’s exact test and Student’s t test or Wilcoxon rank–sum test, respectively. Correlations of EAT with multiple clinical and biochemical variables were performed using Spearman’s correlation analysis. The intraclass correlation coefficient (ICC) was used to assess intra-observer agreement. The ICC for reliability was interpreted as poor (ICC < 0.4), moderate (0.4 ≤ ICC < 0.6), good (0.6 ≤ ICC < 0.8), and excellent (ICC > 0.8). To assess the discriminative ability of epicardial thickness in predicting coronary heart disease, we constructed receiver operating characteristic (ROC) curves. The area under the respective ROC curve (AUROC) was used as a measure of the diagnostic value of the EAT measurement [14].

We considered a *p*-value less than 0.05 as statistically significant. All statistical analyses were performed using the Statistical Package for the Social Sciences (Statistical Package for the Social Sciences, version 20; Armonk, New York, NY, USA) and GraphPad Prism version 9 (La Jolla, CA, USA) software.

## 3. Results

Characteristics of the Study Population

Clinical and laboratory data of all patients with or without CAD are summarized in Table 1.

Upon admission, patients exhibited varying cardiac conditions: stable angina in 46%, unstable angina in 39.3%, ST-elevation acute myocardial infarction (STEMI) in 8.8%, and non-ST-elevation myocardial infarction (NSTEMI) in 5.8%. When assessing the coronary arterial tree, approximately 38% of cases showed no significant stenoses, while 24% had single-vessel disease, 20% had two-vessel disease, and 18% had three-vessel disease (Table 2).

**Figure 1:**
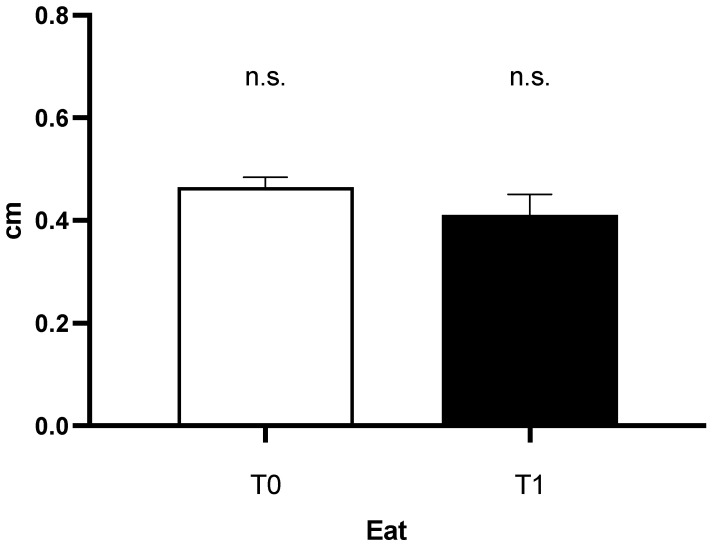
Epicardial fat tissue thickness in the general refence population at enrollment and after three years.

At the beginning of the study (T0), the mean thickness of epicardial fat during end diastole (EAT) it was 0.47 cm (+/− 0.20 SD). Fast forward to the three-year follow-up (T1), the mean EAT was 0.41 cm (+/− 0.18 SD). Interestingly, in the general reference population, no statistically significant differences were observed between EAT at T0-T1 (Figure 1).

**Figure 2:**
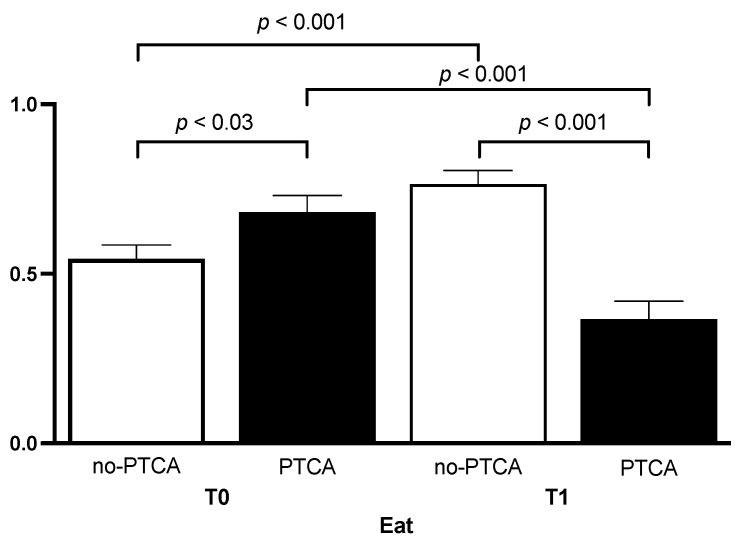
Epicardial fat tissue thickness in patients, categorized by percutaneous transluminal coronary angioplasty (PTCA) at enrollment and after three years, illustrating the dynamic changes in epicardial fat thickness over the specified time period.

Out of the 150 patients who underwent coronary angiography, 60 (40%) underwent percutaneous transluminal coronary angioplasty (PTCA). Subgroup analysis showed a statistically significant differences in EAT between revascularized and non-revascularized patients at T0 (*p* < 0.03; t = 2172, df = 148; Figure 2). Additionally, EAT in patients undergoing PTCA at T1 was significantly lower than EAT at T1 in non-revascularized patients (t = 2.615; df = 88; *p* < 0.001; Figure 2). Notably, there was some differences between EAT group. In the context of non-revascularized groups, EAT at T1 was significantly increased than EAT at T0 (*p* < 0.001), while EAT at T1 in revascularized patients was significantly reduces than EAT at T0 (*p* < 0.001). The intra-observer agreement for measuring echocardiographic EAT was excellent (ICC 0.85; 95% CI 0.72–0.93, *p* < 0.001).

**Figure 3:**
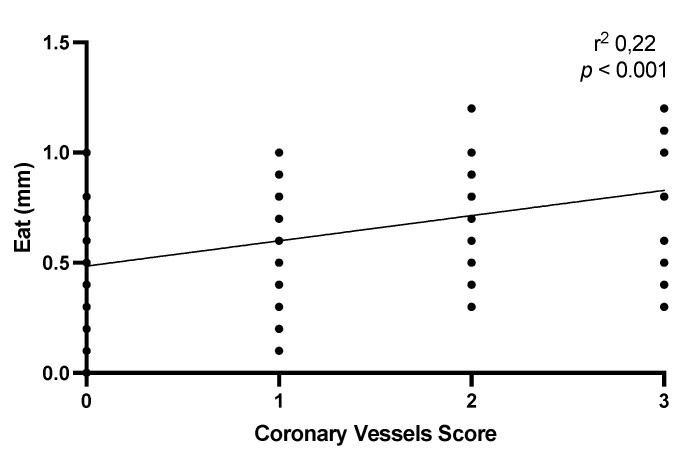
The image illustrates a correlation between epicardial fat thickness and the extent of coronary artery disease at enrollment.

Furthermore, we explore the correlation between EAT and the number of coronary arteries involved at the enrollment. Not surprisingly, the correlation analysis highlighted the presence of a positive and statistically significant correlation between the extent of coronary artery disease, i.e., the number of arteries affected by atherosclerotic pathology, and the thickness of epicardial fat. Specifically, the epicardial fat thickness was greater in patients with triple-vessel coronary artery disease (Figure 3).

**Figure 4:**
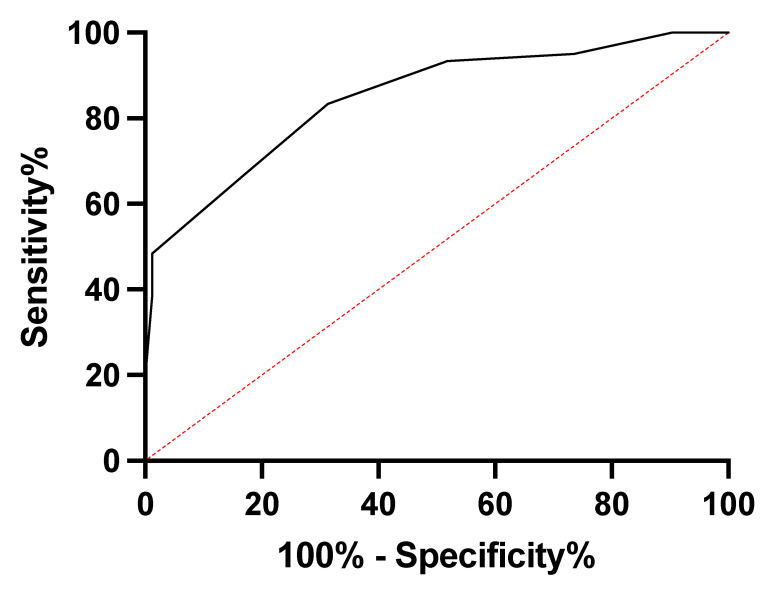
Receiver operating characteristic (ROC) curve depicting the predictive ability of cardiovascular risk based on epicardial fat tissue thickness.

Lastly, the diagnostic utility of EAT in predicting coronary heart disease was assessed using ROC curves, and the area under the curve (AUROC) was 0.84 (95% CI: 0.7847 to 0.9135; *p* < 0.001). Based on these results, a cut-off value of 0.65 cm was identified as the best compromise between sensitivity (72%) and specificity (58%) for predicting cardiovascular risk (Figure 4).

## 4. Discussion

Cardiovascular disease remains a leading cause of morbidity and mortality worldwide, necessitating ongoing research into novel predictors and risk factors for early detection and intervention. Among these, epicardial adipose tissue (EAT) has garnered significant attention due to its intricate relationship with the coronary vasculature and its potential as a predictive biomarker for cardiovascular health.

Our investigation focused on elucidating the role of epicardial fat tissue as a predictor of cardiovascular risk and its clinical relevance in the context of coronary artery disease (CAD). We found compelling evidence that EAT serves as more than just a passive depot of fat; rather, it actively influences the cardiometabolic milieu, contributing to a spectrum of lipotoxic effects collectively known as cardiotoxicity. This discussion provides a comprehensive analysis of our findings, their implications, and their alignment with contemporary research.

The increase in EAT is associated with lipotoxic effects that can detrimentally affect the cardiovascular system. This phenomenon, referred to as cardiotoxicity, encompasses various mechanisms such as inflammation, oxidative stress, and the secretion of adipokines and cytokines, which can lead to endothelial dysfunction and atherosclerosis. The unique characteristics of epicardial fat contribute to its cellular cross talk with cardiac muscle due to the absence of a separation band between epicardial fat and both the myocardium and the adventitia of coronary vessels, as well as the shared microcirculation with the myocardium. Epicardial fat’s variability in cellular components allows for interactions between adipose tissue and the heart [20]. Epicardial fat serves several essential physiological functions in the heart: approximately 70% of the heart’s energy production relies on beta-oxidation of free fatty acids [21]. Epicardial fat plays an active role in energy and lipid homeostasis by directly interacting with coronary arteries. It facilitates the transport of fatty acids to the myocardium, both passively through concentration gradients and facilitated by transport proteins such as FABP-4 (fatty-acid binding protein 4) [22]. Additionally, epicardial fat secretes vasoactive factors that modulate coronary vascular tone. It acts as a buffer, absorbing free fatty acids to create a local energy reserve (lipolysis) and preventing the risk of cardiac lipotoxicity (lipogenesis) [23]. Moreover, epicardial fat can adapt to metabolic conditions. It undergoes trans-differentiation from white to brown adipocytes, providing protection to the heart and coronary arteries against hypothermia and oxidative stress through thermogenesis [4]. Notably, the epicardial fat layer provides an additional layer of mechanical protection, cushioning the heart, and, at the level of the coronary arteries, protecting them against the torsion of arterial pulse waves and cardiac contraction [24]. Lastly, EAT expresses and secretes various adipokines, vasoactive factors, and growth factors. Adipokines, with their endocrine and paracrine properties, play significant roles in both cardioprotection and cardiovascular disease mechanisms. The regulatory mechanisms involved in the production of pro-inflammatory (e.g., TNF-α, IL-1 and MCP-1) and anti-inflammatory (e.g., adiponectin, adrenomedullin and omentin) adipokines are not yet fully understood [25,26]. The dynamic interplay between epicardial fat and the coronary vessels is of particular interest. These two components are in continuous, bi-directional communication with each other, functioning as an integrated system that complements each other in maintaining homeostasis. EAT, through its paracrine and endocrine activities, can influence the surrounding coronary arteries by promoting inflammation and fibrosis, contributing to vascular dysfunction, and ultimately promoting CAD. Epicardial fat has the potential to impact the coronary arteries through multiple pathways, facilitated by the release of various bioactive products. For instance, adipokines, chemokines, gaseous messengers (i.e., hydrogen sulfide and nitric oxide), micro-particles, reactive oxygen species and fatty acids [20,27]. Within the vascular wall, these molecules exert pleiotropic effects on multiple targets, including macrophage activation (promoting proinflammatory phenotype instead of anti-inflammatory phenotype), modulation of oxidative stress (affecting NADPH-oxidase activity and endothelial NO synthase coupling), innate inflammatory response, local activation of endothelial cells, migration of vascular smooth muscle cells (VSMCs), neointima formation, and regulation of vascular tone [28,29].

In line with other recent studies [30], we observed in our study that there is a significant correlation between the thickness of EAT and the severity of CAD. Patients with angiographically significant CAD had thicker EAT compared to those without CAD, underscoring the role of EAT as a marker of visceral adipose tissue in the development and progression of CAD. Our research provides strong support for the utility of EAT measurement, particularly in the end-diastolic phase, as a predictor of cardiovascular risk. We identified a cut-off value of 0.65 cm as the best compromise between sensitivity and specificity, making it a valuable diagnostic tool for risk stratification.

In our study, we measured the diagnostic reliability of echocardiography in assessing EAT and found it to be a feasible and reproducible method. The clinical significance of this measurement is underscored by its ability to identify individuals at higher risk for CAD. This offers the potential for early intervention and more personalized patient care, ultimately leading to improved outcomes.

Furthermore, our investigation also explored the impact of coronary revascularization (percutaneous transluminal coronary angioplasty, PTCA) on EAT thickness. Our findings revealed that EAT decreased sharply significant after coronary revascularization. This reduction in EAT thickness is likely reflective of the positive effects of revascularization on coronary blood flow and overall cardiovascular health. Conversely, in the non-revascularized group, we observed an increase in EAT, which could be attributed to the persistent underlying CAD and its associated metabolic and inflammatory processes.

It is worth noting that our study appears to be the first to comprehensively evaluate the clinical significance of EAT, as measured via echocardiography, in patients with CAD before and after PTCA. This contributes to the growing body of knowledge on the dynamic relationship between EAT and CAD, especially in the context of interventional cardiology.

While our study provides valuable insights into the dynamic relationship between epicardial fat tissue (EAT) and cardiovascular health, it is essential to acknowledge certain limitations that warrant consideration. These limitations may impact the generalizability and interpretation of our findings. Our study relied on echocardiography for the measurement of epicardial fat thickness. It is crucial to recognize that echocardiographic measurements can be operator-dependent, and despite our efforts to maintain consistency by employing a single operator, inherent variability may exist. Future studies incorporating an analysis of inter-operator variability could provide a more comprehensive understanding of the reliability of these measurements. Furthermore, although we measured epicardial fat thickness during the end-diastolic phase, acknowledging its physiological relevance, the dynamic nature of epicardial fat throughout the cardiac cycle may introduce variability. Further investigations exploring the impact of different cardiac phases on epicardial fat measurements could offer additional insights into its behavior. Lastly, our study primarily adopts an observational design, and while we establish associations between epicardial fat thickness, coronary artery disease (CAD), and the effects of percutaneous transluminal coronary angioplasty (PTCA), causal relationships cannot be definitively inferred. Longitudinal studies and randomized controlled trials would be instrumental in establishing causation and elucidating the potential benefits of interventions. Acknowledging these limitations, our study contributes to the existing body of knowledge on the clinical significance of epicardial fat in cardiovascular health. Addressing these limitations in future research endeavors will further refine our understanding of the role of epicardial fat and its implications for risk prediction, intervention strategies, and overall patient care in the context of cardiovascular disease.

In conclusion, our research substantiates the pivotal role of epicardial fat tissue in predicting cardiovascular health. EAT is not merely a bystander in cardiovascular pathophysiology but actively contributes to the development and progression of CAD, encompassing a range of cardiotoxic effects. Our findings highlight the clinical relevance of EAT as a predictor of cardiovascular risk, with an identified cut-off value of 0.65 cm that balances sensitivity and specificity, making it a valuable diagnostic tool for risk assessment.

Furthermore, our study underscores the potential benefits of coronary revascularization, as evidenced by the reduction in EAT thickness after PTCA. This reduction may reflect improved coronary blood flow and overall cardiovascular health, emphasizing the significance of timely intervention.

Incorporating EAT measurements into clinical practice may lead to more precise risk stratification and tailored treatment strategies, ultimately enhancing patient care and reducing the burden of cardiovascular disease. Our research adds to the growing body of evidence supporting the integration of EAT assessment in cardiovascular risk assessment and management, paving the way for improved patient outcomes and the prevention of CAD-related complications. Further studies are warranted to validate and expand upon our findings, potentially leading to even more effective strategies for early detection and intervention in cardiovascular disease.

## Figures and Tables

**Table 1:** Clinical and laboratory data of all patients with or without CAD, providing a comprehensive overview of relevant variables for a thorough understanding of the study cohort.

Baseline Characteristics (N = 150)	
Age, years	67 ± 9
Male	106 (71%)
Waist, Kg	80.25 ± 15.2
BMI, Kg/m^2^	28.9 ± 5.3
Systolic blood pressure, mmHg	123.86 ± 18.8
Diastolic blood pressure, mmHg	73 ± 9
Haemoglobin, g/dL	13.5 ± 1.68
White blood count, mg/dL	8.23 × 10^3^ ± 2.96
Platelet count, mg/dL	222 × 10^3^ ± 78.5
Glycemia, mg/dL	116.78 ± 41.09
HbA1c, %	6.38 ± 1.07
Creatinine, mg/dL	0.99 ± 0.42
Azotemia, mg/dL	5.74 ± 1.58
Triglycerides, mg/dL	125.7 ± 57.71
Total cholesterol, mg/dL	170.34 ± 65.28
LDL cholesterol, mg/dL	103.29 ± 32.55
HDL cholesterol, mg/dL	45.2 ± 12.78
**Medical history**	
Chronic kidney disease	17 (11%)
Dyslipidemia	102 (68%)
Diabetes	54 (36%)
Heart failure	108 (72%)
Previous acute myocardial ischaemia	52 (35%)
Previous stroke	5 (3%)
Arterial hypertension	82 (55%)
Smoking	
*Current*	38 (25%)
*Former*	59 (39%)
*Never*	53(35%)

**Table 2:** Distribution of coronary artery involvement within the study population.

Coronary Vessels Score	
*0* NO-CAD	57 (38%)
*1* Monovasal	36 (24%)
*2* Bivasal	30 (20%)
*3* Trivasal	27 (18%)

## Data Availability

Data is unavailable due to local privacy restrictions.
